# Supplementary material for: Induction of BVR-A Expression by Korean Red Ginseng in Murine Hippocampal Astrocytes: Role of Bilirubin in Mitochondrial Function via the LKB1–SIRT1–ERRα Axis
Source: Antioxidants (Basel). 2022 Sep 1;11(9):1742. doi: 10.3390/antiox11091742 (PMC9496118; doi:10.3390/antiox11091742)
Supplement: Supplementary file 1 [file antioxidants-11-01742-s001.zip › antioxidants-1889193-supplementary.pdf]

Supplementary Figure S1

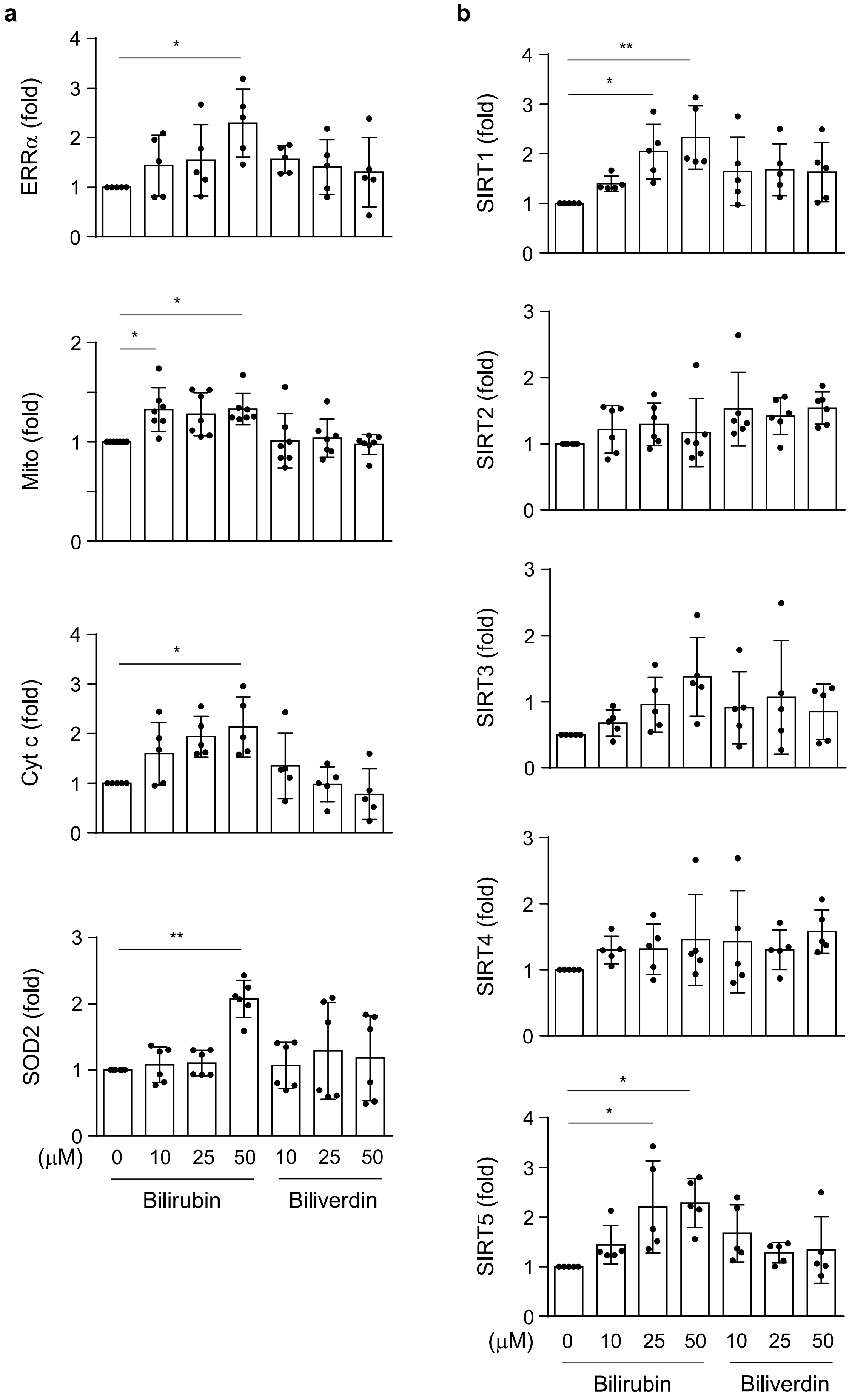

Supplementary Figure S1. (**a-b**) Astrocytes were treated with 50 μM bilirubin or 50 μM biliverdin for 4 h and bands from western blot were analyzed ((*n* = 5-7). **P* < 0.05; ***P* < 0.01.

Supplementary Figure S2

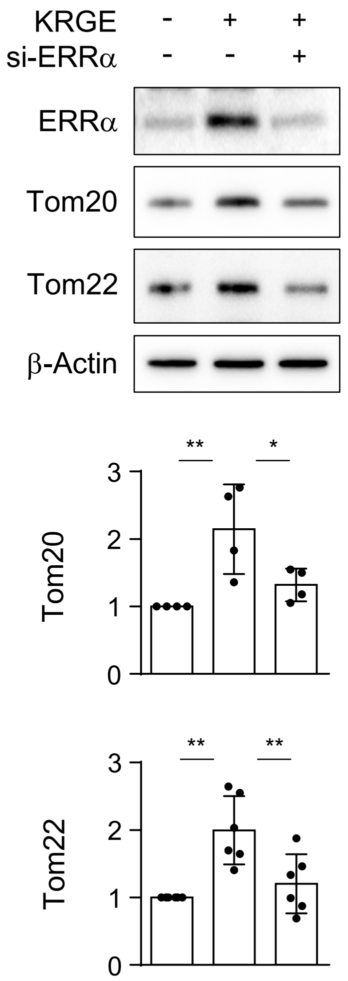

Supplementary Figure S2. Astrocytes were transfected with ERRα siRNA, then treated with KRGE for 24 h. Bands from western blot analysis were quantified (n = 4 or 6). *$P < 0.05$; **$P < 0.01$.
